# Corrosion Properties of the Composite Coatings Formed on PEO Pretreated AlMg3 Aluminum Alloy by Dip-Coating in Polyvinylidene Fluoride-Polytetrafluoroethylene Suspension

**DOI:** 10.3390/polym16202945

**Published:** 2024-10-21

**Authors:** Vladimir S. Egorkin, Igor E. Vyaliy, Andrey S. Gnedenkov, Ulyana V. Kharchenko, Sergey L. Sinebryukhov, Sergey V. Gnedenkov

**Affiliations:** Institute of Chemistry Far Eastern Branch of the Russian Academy of Sciences, Vladivostok 690022, Russia; egorkin@ich.dvo.ru (V.S.E.); asg17@mail.com (A.S.G.); ulyana-kchar@mail.ru (U.V.K.); sls@ich.dvo.ru (S.L.S.); svg21@hotmail.com (S.V.G.)

**Keywords:** aluminum, barrier coatings, plasma electrolytic oxidation, polytetrafluoroethylene, salt fog, corrosion tests

## Abstract

This paper presents the results of an evaluation of corrosion properties of PEO pretreated AlMg3 aluminum alloy samples with polymer coatings obtained by dip-coating in a suspension of superdispersed polytetrafluoroethylene (SPTFE) in a solution of polyvinylidene fluoride (PVDF) in N-methyl-2-pyrrolidone at different PVDF:SPTFE ratios (1:1, 1:3, 1:5, and 1:10). The electrochemical tests showed that samples with a coating formed at a ratio of PVDF to SPTFE of 1:5 possessed the best corrosion properties. The corrosion current density of these samples was more than five orders of magnitude lower than this parameter for bare aluminum alloy. During the 40-day salt spray test (SST) for samples prepared in a suspension at a PVDF:SPTFE ratio of 1:1–1:5, the formation of any pittings or defects was not detected. The PVDF:SPTFE 1:5 sample demonstrated, as a result of the 40-day SST, an increase in corrosion current density of less than an order of magnitude. The evolution of the protective properties of the studied samples was assessed by a two-year field atmospheric corrosion test on the coast of the Sea of Japan. It was revealed that the samples with the PVDF:SPTFE 1:5 coating had electrochemical parameters that remained consistently high throughout the one year of exposure. After this period, the polymer layer was destroyed, which led to a deterioration in the protective characteristics of the coatings.

## 1. Introduction

According to [1], the cost of corrosion in industrialized countries is about 3–4% of their gross domestic product, making uncontrolled corrosion destruction a global concern [2]. Therefore, the corrosion protection of metals and alloys against aggressive atmospheres by preventing or slowing down the corrosion process is of great importance [3,4].

The acceleration of corrosion processes in the marine atmosphere is driven by high salinity, increased humidity, and ultraviolet radiation [5]. Therefore, marine industry objects have to be properly protected [6,7]. Because of this, technologies for forming different coatings on metals and alloys are intended to protect them against corrosion [8]. Polymer coatings can provide such protection. They are at the forefront of anticorrosion protection since, in most cases, they form a low permeable film for corrosive environments and possess mechanical durability and high chemical inertness. One of the best polymers for the formation of such coatings is polytetrafluoroethylene (PTFE) [9]. The molecular structure of PTFE ensures stable anticorrosion and good mechanical properties, demonstrating low values of surface energy and coefficients of friction [10,11,12]. On the other hand, high chemical inertness due to such a structure of PTFE leads to the fact that uniform distribution of the polymer film over the metal surface is a very difficult task.

There are methods to improve the quality of formed polymer coatings by using PTFE microparticle and nanoparticle suspensions [13], or nano-sized particles of PTFE can be applied to metal under the influence of electrophoretic deposition [14]. Also, a variety of techniques, such as hot filament chemical vapor deposition [15], physical vapor deposition [16], and sputtering [17], have been used to deposit the polymer atop the metals and alloys. Moreover, some methods for better adhesion of PTFE to a metal substrate require the use of perfluorooctanoic acid, which is very harmful (toxic) to the environment and living organisms. Often, a high processing temperature is necessary to form a solid polymer film. The processing of PTFE at elevated temperatures frequently promotes the release of quite harmful compounds.

Therefore, there is a growing trend toward the application of PTFE-containing layers without the necessity of high-temperature treatment. In the research of Lu et al. [18], a composite coating including PTFE nanoparticles was formed on 2024 aluminum alloy at a minimum temperature of 80 °C. A high adhesion, density, and thickness of the polymer film were achieved through vacuum impregnation of polytetrafluoroethylene into the outer porous layer of the ceramic coating formed by PEO [19,20]. Meng et al. [21] prepared a cross-linked waterborne acrylic/PTFE composite coating, which was dried at room temperature.

In this work, we propose a way that is simpler and does not involve the stage of the high-temperature treatment of the superdispersed polytetrafluoroethylene (SPTFE) microparticles (spherical particles composed of nanosheets obtained using the gas-dynamic thermal destruction method [22]), but in their binding with polyvinylidene fluoride (PVDF) and the formation of a dense composite layer by application of this polymer mixture into a PEO coating structure. PVDF has been widely used as a binding component for obtaining films containing micro/nanoparticles of inorganic and organic materials [23,24,25,26,27]. Our previous investigations showed that when its solution is modified with SPTFE powder, the pores of PEO coating are sealed with polymer. Moreover, a multi-level relief is built on the surface of the forming composite coatings because of smaller microparticles, which stick together on the surface of the sample during the PVDF. Thus, the high-temperature treatment stage is eliminated, which ensures environmental safety and facilitates the technology.

Previous research on aluminum alloy with polyvinylidene fluoride-polytetrafluoroethylene coatings has established low wettability and high barrier and anti-icing properties of such layers [28,29]. Salt spray tests [30,31,32,33,34,35], as well as long-term electrochemical tests in a 3.5 wt.% NaCl solution [36,37,38], are most often used in order to assess the protective properties of the different types of anticorrosion coatings. Carrying out field corrosion tests allows us to obtain accurate data on anticorrosion characteristics of protective coatings and their service life in specific operating conditions. The maximum effect and reliability of results can be achieved by the application of these methods in a complex study.

However, such complex studies of the anticorrosion properties of polymer-containing coatings carried out in laboratory and natural conditions are not common at the moment. For this purpose, this study assessed the protective properties of composite coatings by complex electrochemical testing of the samples before and after their exposure to 40 days of salt spray test and 3 and 6 months, as well as 1 and 2 years, of atmosphere corrosion.

## 2. Materials and Methods

### 2.1. Parameters of the Samples and Its Treatment

Plasma electrolytic oxidation of AlMg3 (wt.%: Mg—3.75; Si—0.78; Fe—0.43; Mn—0.38; Ti, Cu, Zn—up to 0.1 each; Cr—0.05, Al—balance) aluminum alloy (Kamensk-Uralsky Metallurgical Plant OJSC, Kamensk-Uralsky, Russia) samples with size of 50 × 50 × 2 (mm)^3^ was carried out in bipolar mode for 15 min. Previously, the Al sheets were ground with silicon carbide sandpaper in succession from 320 up to 1200 grit, then washed with deionized water, and dried in the Binder FD53 oven (BINDER GmbH, Tuttlingen, Germany) for 4–5 min at (100 ± 5) °C. PEO was carried out in bipolar polarization mode with a frequency of 300 Hz for 15 min. The voltage during the anodic period was increased potentiodynamically from 30 to 540 V at a sweep rate of 8.5 V∙s^−1^; then, the voltage value was fixed at 540 V for 14 min. During the cathodic period, current density was galvanostatically maintained at 0.12 A∙cm^−2^. The duty cycle was equal to 1. An aqueous solution of 20 g/L Na_2_SiO_3_∙5H_2_O, 10 g/L Na_2_B_4_O_7_∙10H_2_O, 2 g/L NaF, and 2 g/L KOH was used as an electrolyte.

PEO pretreated samples (see Table 1) were coated with a polymer film by dip-coating in a suspension of superdispersed polytetrafluoroethylene (SPTFE) (Vladivostok, Russia, ^®^Forum) in 6% solution of polyvinylidene fluoride −(C_2_H_2_F_2_)_n_− (Shandong Hengyi New Material Technology Co., Ltd., Zibo, Shandong Province, China) in N-methyl-2-pyrrolidone (C_5_H_9_NO)_n_ (Merck, Darmstadt, Germany). In this study, the ratio of PVDF:SPTFE in the suspension was equal to 1:1, 1:2, 1:3, 1:4, 1:5, and 1:10. After dip-coating, the specimens were dried for 3 h in a FD532 drying oven (BINDER GmbH, Tuttlingen, Germany) at 65 °C.

### 2.2. Surface and Structure Characterization

The morphology structure of the samples was investigated by scanning electron microscopy (SEM). SEM images of the sample were obtained using a Zeiss Marlin scanning electron microscope (Carl Zeiss Group, Oberkochen, Germany) with a Silicon Drift Detector X-Max^N^ 80 (Oxford Instruments plc, Abingdon, UK).

The EDS data were obtained on SEM of the Federal Scientific Center for East Asian Terrestrial Biodiversity of the Far Eastern Branch of the Russian Academy of Sciences (Vladivostok, Russia).

The 3D surface profiles of the samples were carried out using open-source software for image analysis and processing, ImageJ v1.52 (National Institutes of Health, Bethesda, MD, USA).

Cross-sections of the formed coatings were prepared by cold mounting the specimen in a 30 mm cup using an Epovac impregnator (Struers A/S, Copenhagen, Denmark) and ViaFix acrylic resin. The samples were polished on a Tegran 25 device (Struers A/S, Copenhagen, Denmark) using an MD-Largo disk with a 9 μm diamond suspension and then using MD-Mol with 3 μm diamond suspensions. The MD-Chem disk with 0.04 μm non-drying colloidal silica suspension (OP-U) for final polishing was used. After polishing, the product was washed for 30 min in deionized water using an ultrasonic bath Bandelin Sonorex Super RK 100 (BANDELIN Electronic GmbH & Co. KG, Berlin, Germany) and dried in warm air.

### 2.3. Electrochemical Test

Electrochemical properties were investigated using ModuLab XM ECS electrochemical test system (AMETEK Scientific Instruments, Wokingham, UK) and XM studio ECS v 3.4 software. Measurements were carried out in a three-electrode cell in a 3.5 wt.% NaCl. A platinum mesh was used as a counter electrode, and a saturated calomel electrode was used as a reference electrode. The exposed area of samples was equal to 1 cm^2^.

The samples were kept in a solution for 60 min prior to the electrochemical tests to achieve a steady state. The sinusoidal signal with an amplitude of 10 mV (rms) was used for impedance measurements. The experiments were carried out in the frequency range from 0.1 MHz to 0.01 Hz at a logarithmic sweep of 10 points per decade. The potentiodynamic polarization was carried out at a sweep rate of 1 mV∙s^−1^ in the range from *E*_C_ − 0.25 V to *E*_C_ + 1.5 V, where *E*_C_ is the corrosion potential. The fitting of experimental data to the Butler–Volmer equation was carried out using the Levenberg–Marquardt approach, which allows us to calculate parameters such as corrosion potential *E*_C_ and corrosion current density *I*_C_. The polarization resistance *R*_p_ = Δ*E*/Δ*j* was determined in a separate experiment during a potentiodynamic polarization test in the range of *E*_C_ ± 20 mV, where a linear dependence of the potential on the current density was observed.

To simulate charge transfer processes at the electrode–electrolyte interface, a parallel *R*–*CPE* circuit was used for the sample without coatings, and an electrical equivalent circuit (EEC) with two series-parallel *R*–*CPE* circuits was used for the samples with coatings. Instead of an ideal capacitance, a constant phase element (*CPE*) was used in this study. The need for such a replacement was due to the heterogeneity of the studied objects. The *CPE* impedance was calculated according to the following equation: *Z*_CPE_ = 1/*Q*(*jω*)^n^, where *ω* is the angular frequency (*ω* = 2*πf*), *j* is the imaginary unit, *n* is the exponential coefficient, and *Q* is a frequency-independent constant. Impedance spectra are presented with experimental data (marked with symbols on the graphs) and lines of connected points, which are calculated parameters of EEC. The accuracy of modeling experimental results is at the error level χ^2^ of ~10^−4^.

### 2.4. Salt Spray Test

The SST was carried out in the salt spray chamber S120 (Ascott Analytical Equipment Ltd., Tamworth, UK) according to ISO 9227:2022 [39]. Corrosion testing was conducted by spraying the neutral 5 wt.% NaCl solution for 40 days.

### 2.5. Corrosion Test in Marine Atmosphere

To study the dynamics of changes in electrochemical properties during atmospheric corrosion, the samples were tested at the Marine Corrosion Exposure Station of the Institute of Chemistry FEB RAS, located on Russkiy Island, Rynda Bay, in the Sea of Japan [40]. Samples with a size of 30 × 30 × 2 (mm)^3^ were exposed at an angle of 45° to the horizon on racks located about 20 m from the coastline. The electrochemical properties of the samples were studied after 3, 6, 12, and 24 months of atmosphere corrosion testing.

## 3. Results

### 3.1. Study of Initial Samples

**Figure 1:**
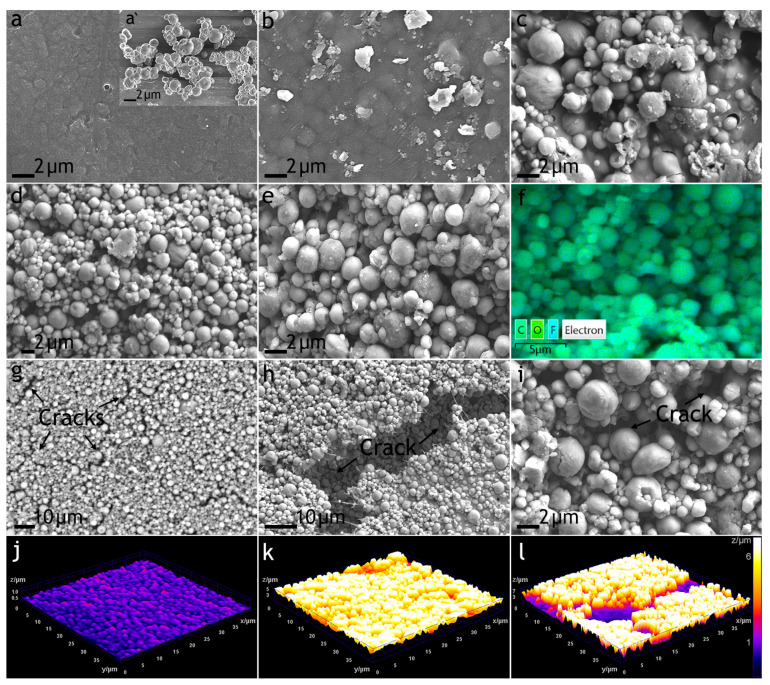
SEM images of the PEO/PVDF sample (**a**); microparticles STTFE (**a’**); and formed coatings by mixing in the ratio of PVDF:SPTFE: 1:1 (**b**), 1:3 (**c**), 1:5 (**d**–**f**), and 1:10 (**g**–**i**); EDS mapping (**f**) the carbon, oxygen, and fluorine on the surface of the PVDF:SPTFE 1:5 sample; 3D surface profiles of the PEO/PVDF (**j**), PVDF:SPTFE 1:5 (**k**), and PVDF:SPTFE 1:10 (**l**) samples.

Analysis of SEM images of the initial PEO/PVDF sample (Figure 1) indicates that its surface is smooth and pores are filled with solidified PVDF, which provides the best adhesion to the PEO coating (Figure 1a). SEM images of the superdispersed polytetrafluoroethylene particles with a diameter of 1–2 µm are shown in Figure 1a’.

The study of the surface morphology of coatings formed in the suspension of fluoropolymers showed that a ratio of 1:1 did not contribute to the increase in the surface roughness of the PVDF film. Apparently, there were too few microparticles to reach the surface of the coatings during solvent evaporation; it is probable that the microparticles remained inside the PVDF layer (Figure 1b). When the concentration of SPTFE microparticles in a PVDF solution increased to a ratio of 1:3, significantly more agglomeration of particles was observed; however, they were not yet tightly combined with each other (Figure 1c). Increasing the ratio of PVDF to SPTFE to 1:5, the composite coating was characterized by very evenly dispersed agglomerates (Figure 1d,e). According to the EDS analysis, the particles were composed of carbon and fluorine (Figure 1f). The microparticles formed large agglomerates, which embedded into PVDF layers and sealed the pores of the PEO coating. At a ratio of more than 1:5, the composite polymer layer was very susceptible to cracking (Figure 1g–i). At the same time, Figure 1j–l show damage to the PVDF:SPTFE 1:10 sample, as well as the defect-free surface of the PVDF film without SPTFE and the modified SPTFE microparticles at a 1:5 ratio.

Analysis of 3D surface profiles shows that with an increase in the composite coating of the SPTFE fraction, the composite coatings had a more developed surface (z increased from 0.7 µm to 7 µm). However, when reaching 1:10, cracks were observed in the coatings, the depth of which could reach 5 µm, which is a critical factor for protective properties.

**Figure 2:**
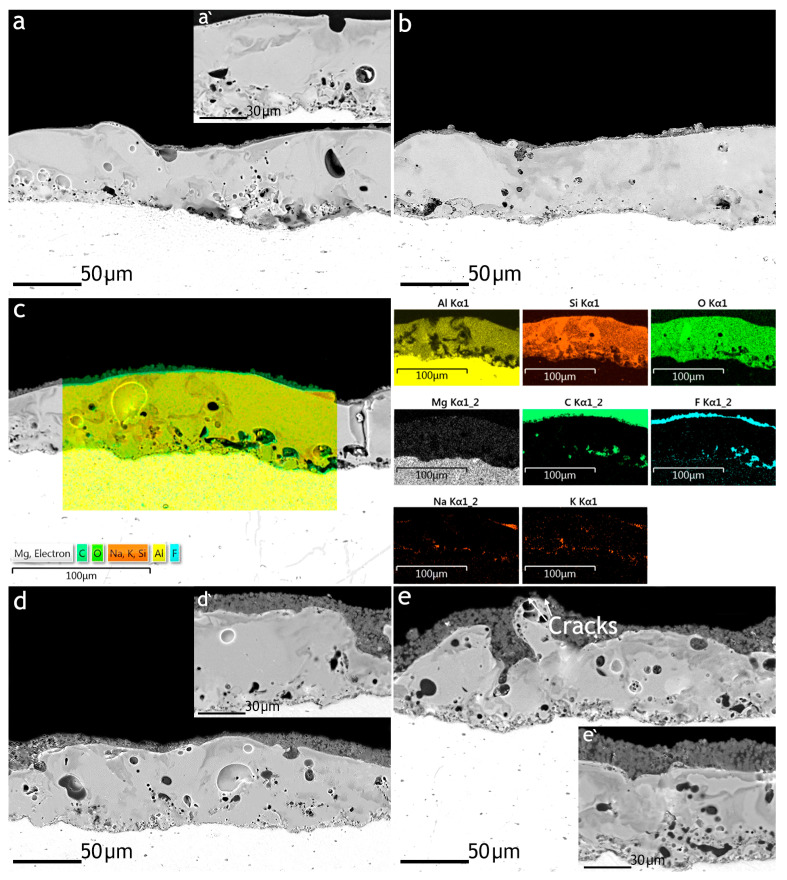
SEM images of the cross-sections of the PEO (**a’**) and PEO/PVDF (**a**) samples and formed coatings in suspension of the SPTFE in the PVDF solution in the ratio of PVDF:SPTFE: 1:1 (**b**), 1:3 (**c**), 1:5 (**d**,**d’**), and 1:10 (**e**,**e’**) and EDS mapping on the cross-sections of the PVDF:SPTFE 1:3 sample (**c**).

Cross-sections of the studied samples are presented in Figure 2. The PEO coating had a thickness of (63 ± 3) μm, porosity of (7 ± 2)%, and a developed surface for strong adhesion of the deposited polymer film. After treatment of the PEO-coated samples in the PVDF solution and SPTFE suspensions, a more uniform formation of the polymer film over the PEO coating was observed (Figure 2a’) since an increase in the ratio of PVDF: SPTFE from 1:1 to 1:10 led to an increase in its thickness (up to (18.1 ± 1.7) µm) compared to the only-PVDF layer ((1.9 ± 0.7) µm).

EDS mapping of the element distribution within the coating thickness showed that the main elements of the PEO layer are aluminum, oxygen, and silicon (Figure 2c). These elements can be included in compounds such as Al_2_O_3_ and SiO_2_ because of the reaction of the aluminum of the substrate with the dissociation products to a greater extent, Na_2_SiO_3_∙5H_2_O (pK_a_ = 9.50) than Na_2_B_4_O_7_∙10H_2_O (pK_a_ = 3.74). EDS analysis of films deposited atop the PEO coating revealed a high content of carbon and fluorine, of which the polymers used are composed.

**Figure 3:**
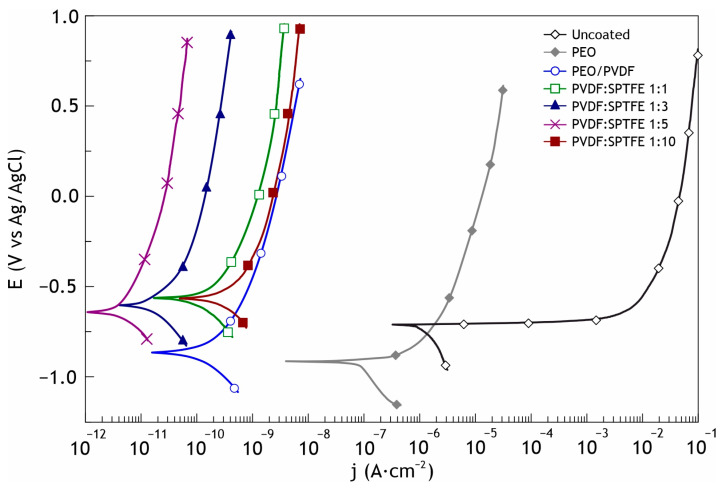
Potentiodynamic polarization curves obtained in 3.5 wt.% NaCl for AlMg3 aluminum alloy with PEO and composite coatings.

The potentiodynamic polarization curves of the bare aluminum alloy and the coated samples are shown in Figure 3. The calculated values of electrochemical parameters are presented in Table 2. In the examined range of potentials, the curve for the uncoated AlMg3 has a form that is characteristic of this alloy; after the cathodic part of the curve, a breakdown of the natural oxide/hydroxide film occurs near the corrosion potential with a corresponding sharp increase in the current density. Polarization curves obtained for the coated samples are located in the zone of substantially lower currents than the curve for bare alloy and exhibit significant inhibition of both anodic and cathodic reactions.

As a result of the assessment of the electrochemical characteristics of the samples, it was revealed that formed protective layers significantly decreased the corrosion current density for samples with composite coatings. This parameter decreased by almost 4.5 orders of magnitude for the PVDF:SPTFE 1:1 sample (*I*_C_ = 7.2 × 10^−11^ A∙cm^−2^) compared to the uncoated aluminum alloy (*I*_C_ = 1.1 × 10^−6^ A∙cm^−2^). The changes in the morphology of the coatings shown in Figure 1 with an increase in the PVDF:SPTFE ratio from 1:1 to 1:5 provided an even higher level of barrier properties (*I*_C_ = 7.5 × 10^−12^ A∙cm^−2^). A further increase in the PVDF:SPTFE ratio to 1:10 led to defects in the polymer layer through which the corrosive medium penetrates the substrate (*I*_C_ = 3.9 × 10^−10^ A∙cm^−2^) (Figure 3, Table 2).

**Figure 4:**
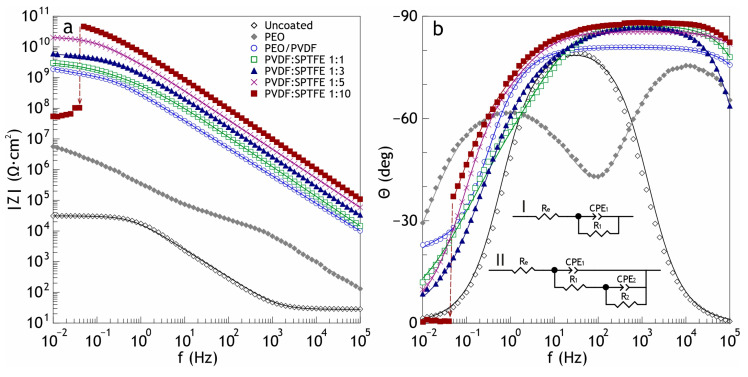
Bode plot (the impedance modulus (**a**) and the phase angle vs. frequency (**b**)) for studied samples in 3.5 wt.% NaCl (I and II in Figure 4b are electrical equivalent circuits for fitting the experimental data of the uncoated sample and with coatings, respectively).

Figure 4 shows the results of impedance measurements in the Bode plots (the impedance modulus |Z| and the phase angle vs frequency). The spectra, similar to the polarization curves for samples with composite coatings, confirm the high barrier properties. The increase in |Z| at the lowest frequency in a series of studied samples is highest for PVDF:SPTFE 1:5, which was six orders of magnitude more than the uncoated aluminum sample (2.9 × 10^4^ Ω∙cm^2^). The values of the phase angle *Θ* for the coated samples exhibited capacitive behavior and had a tendency to decrease to –90° at high and middle frequencies with an increasing PVDF:SPTFE ratio (Figure 4b).

For the PVDF:SPTFE 1:10 sample, a sharp decrease by almost three orders of magnitude at low frequencies in the impedance modulus occurred (Table 3), and the phase angle *Θ* ≈ 0°.

**Figure 5:**
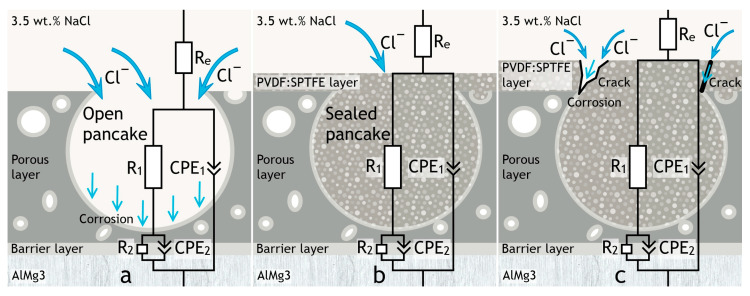
Simulations of aggressive environment penetration through barrier layers to the substrate during electrochemical testing of the PEO (**a**), PVDF:SPTFE 1:5 (**b**), and PVDF:SPTFE 1:10 (**c**) samples.

A sharp decrease in the electrochemical parameters for a coating with a multi-level relief (PVDF:SPTFE 1:10 sample) occurs because of the accelerated penetration of chloride anions from the electrolyte into the pores of the PEO layer and causes destruction of the aluminum substrate. The simulation of the influence of the polymer composition on the corrosion properties may be provided by using the equivalent electrical circuit and assessing the calculated parameters. Penetration of the corrosive medium to the substrate depends on the morphology of the polymer layer formed atop the PEO coating. Cracks in the polymer coating are the most probable penetration pathways for the corrosion species (see Figure 5). Based on this, by means of electron microscopy and electrochemical research, we determined the optimal PVDF:SPTFE ratio in the applied suspensions is equal to 1:5.

Therefore, for further corrosion tests, the samples with composite coatings formed at a PVDF:SPTFE ratio from 1:1 to 1:5 were taken. Also, to compare the results of uncoated AlMg3, PEO and PEO/PVDF samples were tested.

### 3.2. Salt Spray Test Result

For testing according to ISO 9227:2022 [39], plates of AlMg3 aluminum alloy with dimensions of 5 × 5 × 0.2 cm^3^ were prepared. A part of the tested samples was extracted after 4, 10, 20, 30, and 40 days for investigation.

**Figure 6:**
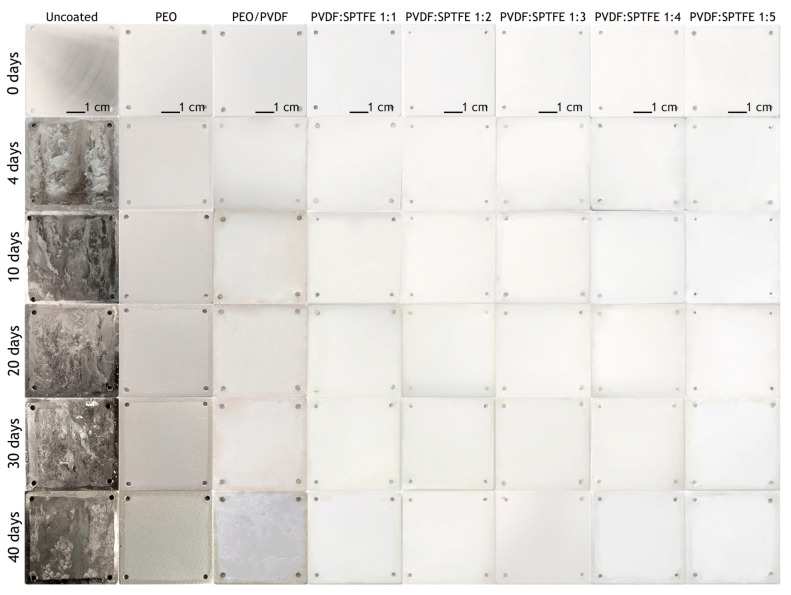
Photographs of the AlMg3 aluminum alloy and samples with PEO and composite coatings before and after salt spray testing for 4, 10, 20, 30, and 40 days.

The most susceptible-to-corrosion samples of the AlMg3 without a coating and with a PEO layer, after being removed from the salt fog chamber, were first placed in a heating chamber to prevent further destruction, and then, they were photographed. Samples with composite coatings were photographed immediately after being removed from the corrosion chamber. Figure 6 shows photographs of the test samples throughout the entire exposure to an aggressive environment.

**Figure 7:**
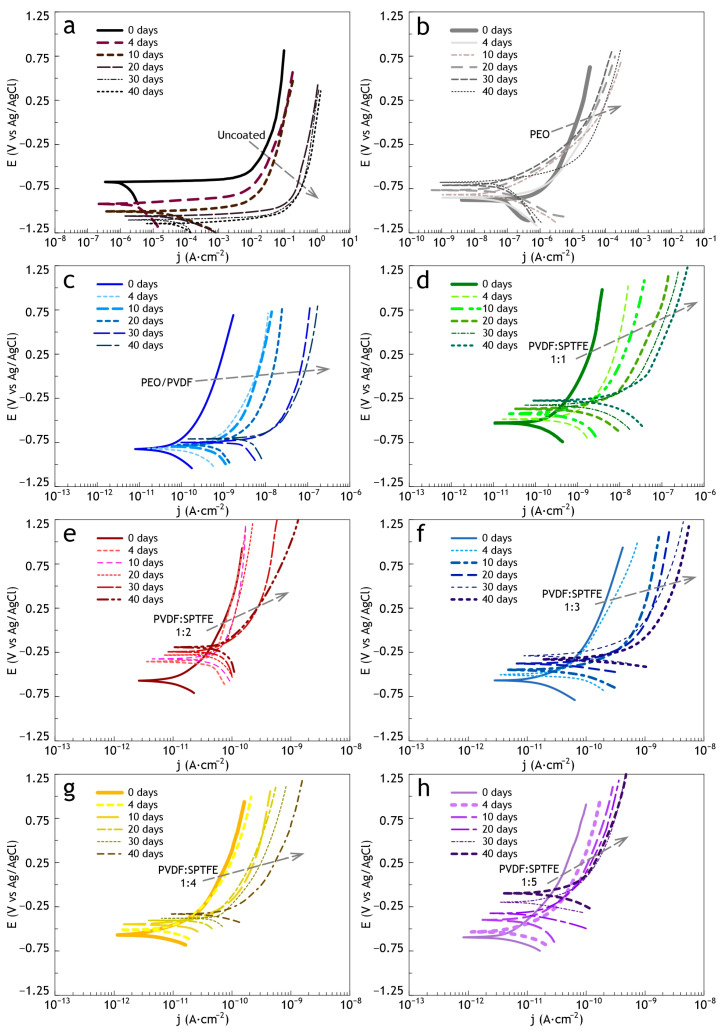
Evolution of the potentiodynamic polarization curves for uncoated AlMg3 aluminum alloy (**a**) and PEO (**b**), PEO/PVDF (**c**), PVDF:SPTFE samples: 1:1 (**d**), 1:2 (**e**), 1:3 (**f**), 1:4 (**g**) and 1:5 (**h**) during salt spray testing.

**Figure 8:**
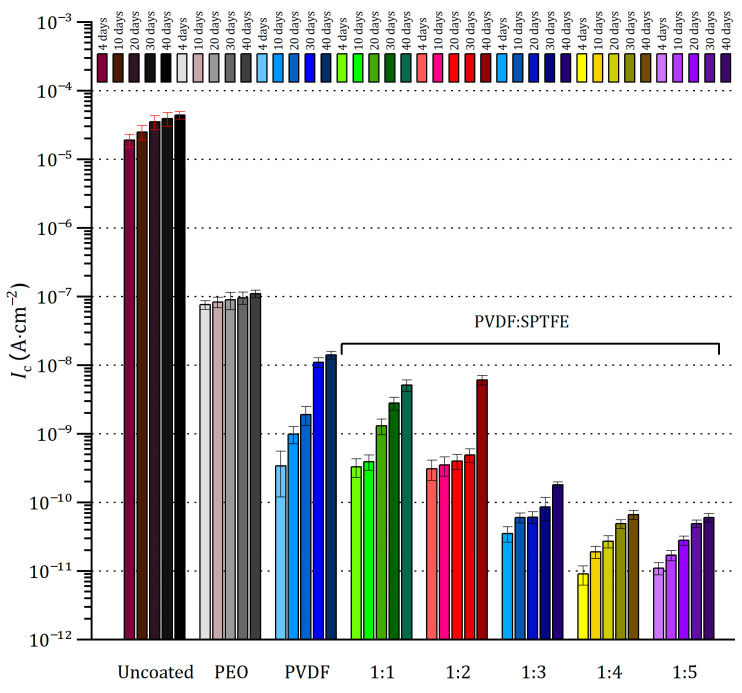
Evolution of corrosion current density during salt spray testing of the uncoated AlMg3 aluminum alloy and with PEO- and composite coatings.

**Figure 9:**
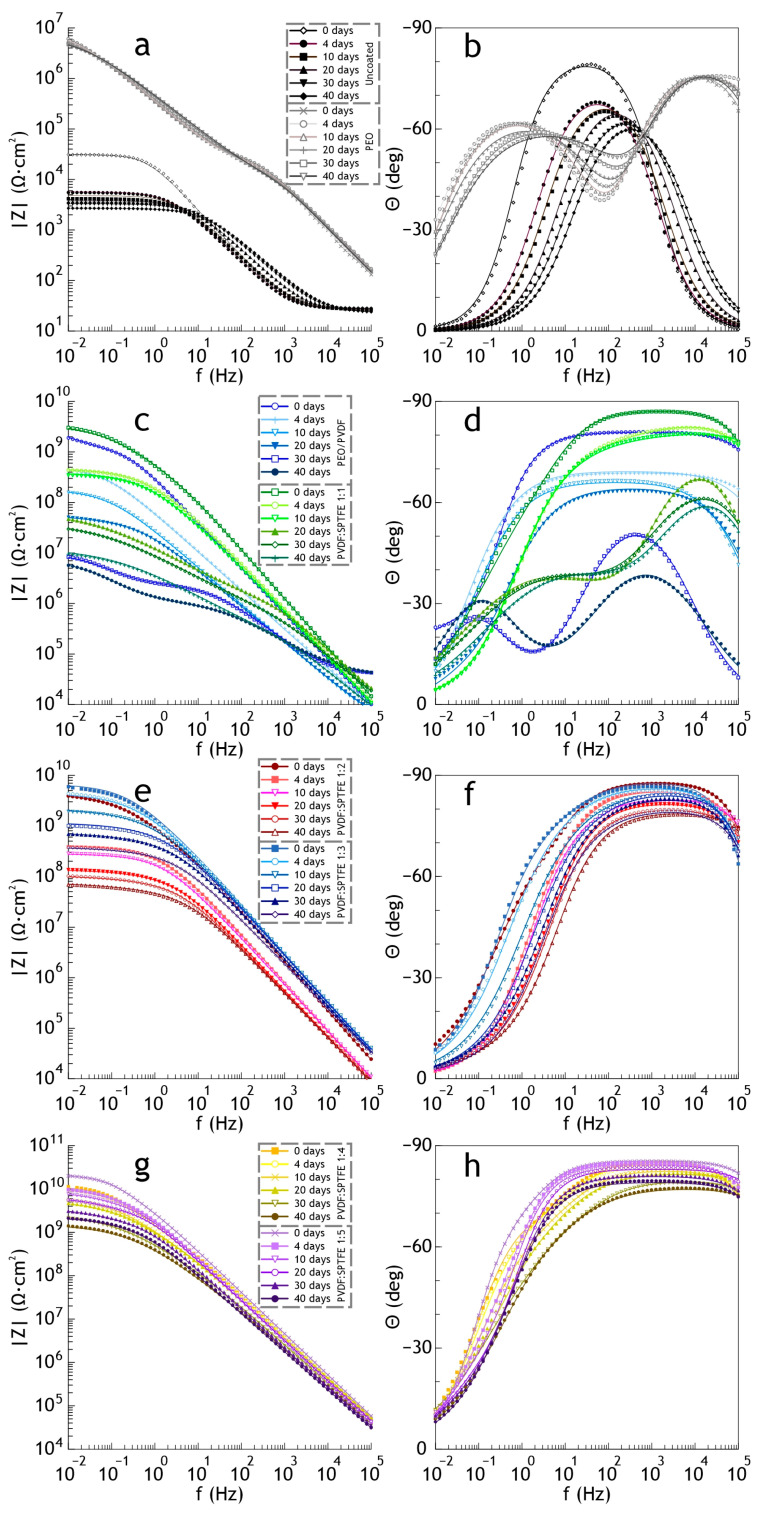
Evolution of the impedance spectra during salt spray testing for AlMg3 aluminum alloy and PEO (**a**,**b**), PEO/PVDF and PVDF:SPTFE 1:1 (**c**,**d**), PVDF:SPTFE 1:2 and 1:3 (**e**,**f**), PVDF:SPTFE 1:4 and 1:5 (**g**,**h**) samples.

**Figure 10:**
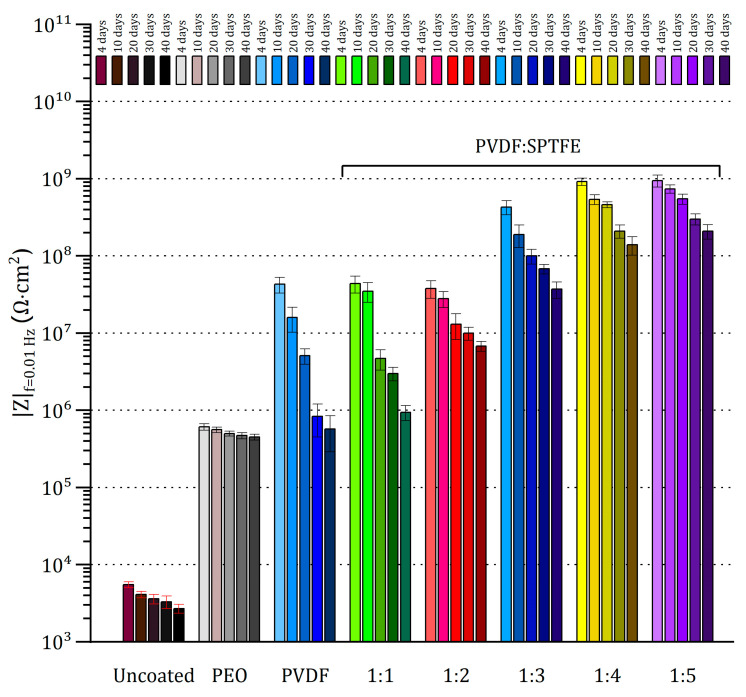
The evolution of impedance modulus measured at 0.01 Hz during salt spray testing of the uncoated AlMg3 aluminum alloy and samples with PEO and composite coatings.

The uncoated sample was significantly corroded after 4 days of exposure (Figure 6). Further exposure led to the appearance of pittings and a significant increase in the amount of the corrosion products deposition. The evolution of electrochemical properties of samples with exposure time from 4 to 40 days is presented in Figure 7 and Figure 8 as potentiodynamic polarization curves, diagram of *I*_C_ values and in Figure 9 and Figure 10 as Bode plots, diagram of |Z| values, respectively. For an uncoated aluminum alloy after 40 days of exposure to salt fog, the corrosion current density increased from 1.1 × 10^−6^ to 4.4 × 10^−5^ A·cm^−2^ (Figure 8, Table S1, Supplementary Material). The measurement of the impedance modulus showed a slight decrease in the resistance of unprotected aluminum alloy samples (2.7 × 10^4^ Ω·cm^2^) (Figure 10, Table S2) in comparison with this parameter before testing (2.9 × 10^4^ Ω·cm^2^). However, prolonged exposure to a corrosive environment led to strong corrosive destruction. 

Analysis of the presented photographs of the appearance of the coatings indicates that aluminum samples protected with a PEO layer were significantly less subject to corrosion. However, after 30 days of salt spray testing, darkening of their surface was observed, which indicates the penetration of the saline solution into the pores and the occurrence of a corrosion process activated in the coating.

At the same time, analysis of polarization curves shows that testing the PEO sample significantly increases the corrosion potential (from −0.87 to −0.67 V (Ag/AgCl)). This indirectly indicates the appearance of the effect of corrosion inhibition due to the filling of the porous part of the coating with the resulting corrosion products. Probably, because of this, further testing up to 40 days led to greater darkening of the PEO film; however, no pitting or peeling of the coating from the substrate was observed (Figure 6). This is also indicated by the values of corrosion current density (Figure 7a and Figure 8) and impedance modulus (Figure 9a and Figure 10) after 40 days of testing the PEO layer, which changed by 1.3 times compared to the initial values of these parameters (8.4 × 10^−8^ A·cm^−2^ and 5.6 × 10^6^ Ω·cm^2^, respectively).

The PEO/PVDF sample showed localized darkening at the end of the test (Figure 6). The low thickness of PVDF film probably resulted in the penetration of the corrosive medium through the PEO coating to the substrate. According to Figure 7b, the PVDF layer provided only one order of magnitude lower corrosion current density than for a sample with a PEO coating (1.1 × 10^−7^ A·cm^−2^) and 2.5 orders of magnitude lower than for an uncoated aluminum alloy (4.4 × 10^−5^ A·cm^−2^). Also, the impedance modulus for the PEO/PVDF coating decreased by 2.5 orders of magnitude (5.7 × 10^6^ Ω·cm^2^) compared to this parameter for the sample before testing (Figure 9 and Figure 10).

The highest barrier properties were demonstrated by the PVDF:SPTFE 1:1–1:5 samples (Figure 8). Photographs of these samples did not reveal any color changes or defects (Figure 6).

Therefore, in the series of PVDF:SPTFE 1:3–1:5 samples, the latter has better corrosive properties with *I*_C_ lower by more than five orders of magnitude (up to 7.5 × 10^−12^ A·cm^−2^) compared to the uncoated aluminum alloy (Figure 8). Analysis of diagrams in Figure 8 and Figure 10 for the PVDF:SPTFE 1:5 sample shows that within 40 days of sample testing, the level of corrosion current density increased by less than an order of magnitude with a corresponding decrease in the level of electrical resistance compared to the initial composite layer.

In general, for all samples, increasing the exposure time in a salt fog environment led to a worsening in electrochemical parameters. Since, in a series of composite coatings, the greatest and least decreasing corrosion resistance was recorded for PEO/PVDF and PVDF:SPTFE 1:5 samples, respectively, these samples were also tested for atmospheric corrosion.

### 3.3. Marine Atmosphere Corrosion Tests

The two-year atmosphere corrosion testing of samples began in the winter (December), and some of the samples were taken out after 3 and 6 months and 1 and 2 years for an intermediate assessment of changes in morphology and electrochemical properties.

**Figure 11:**
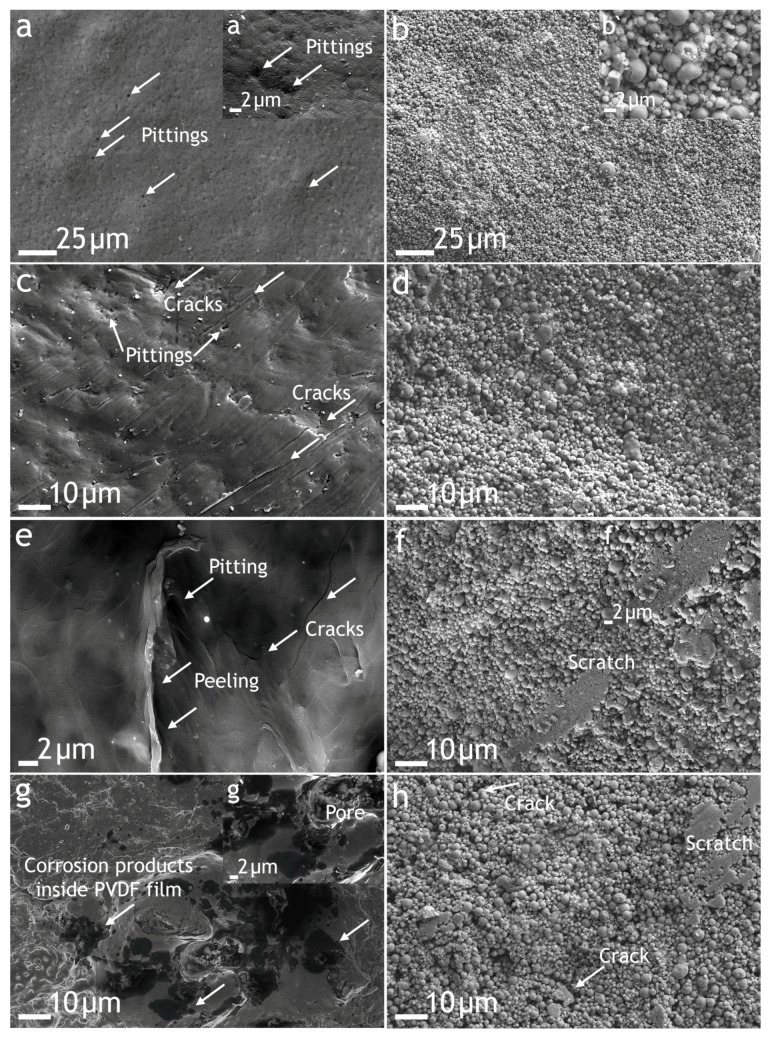
SEM images of the surface PEO/PVDF and PVDF:SPTFE 1:5 samples after 3 months (**a**,**a’**,**b**,**b’**), 6 months (**c**,**d**), 1 year (**e**,**f**,**f’**), and 2 years (**g**,**g’**,**h**) atmosphere corrosion testing.

Analysis of SEM images (Figure 11a) shows that after 3 months (December–February) of exposure on the PEO/PVDF sample, the pittings were seen. For the PVDF:SPTFE 1:5 samples, no pittings occurred during the winter period (Figure 11b,b’).

Subsequent tests of samples resulted in the appearance of microcracks and pitting in the PVDF film (Figure 11c). The PVDF:SPTFE 1:5 sample did not change significantly (Figure 11d).

After 1 year of exposure, additional defects (peeling and scratches) were detected on the samples at the beginning of the new winter period. These defects were more significant for the PEO/PVDF (Figure 11e) than for the PVDF:SPTFE 1:5 sample (Figure 11f).

Consequently, the subsequent exposure of the samples during the second year showed significantly greater corrosion damage in the base PEO coating due to both peeling and aging under the influence of solar ultraviolet of polymer films, regardless of the content of SPTFE microparticles. In the first case, the corrosion process was observed mainly at the boundary of the PVDF film peeling, but corrosion was fixed also in the deep pores of the PEO layer (marked on the micrograph in Figure 11g’). In the second group, scratches and microcracks of the polymer film were also observed, but in a much lesser number (Figure 11h). Thus, the protective properties of composite coatings were reduced, but all defects were superficial, and the PEO layer, which functions as a primer film for the aluminum substrate, remained without critical damage (Figure 11g,h).

**Figure 12:**
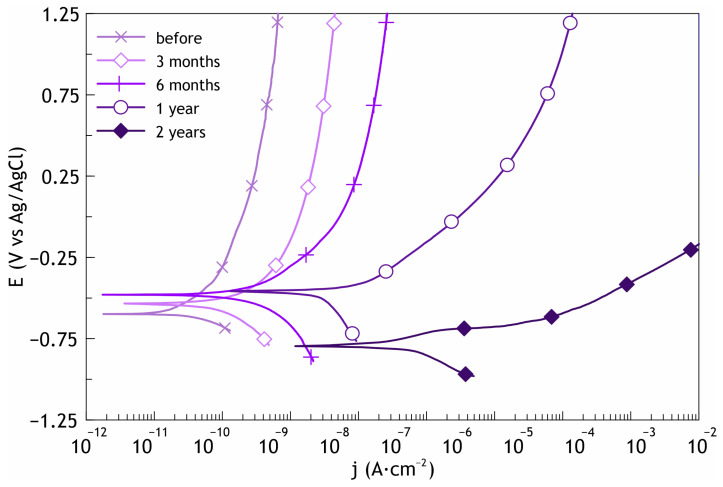
Polarization curves for PVDF:SPTFE 1:5 sample after 3 months, 6 months, 1 year, and 2 years of the atmosphere corrosion testing.

**Figure 13:**
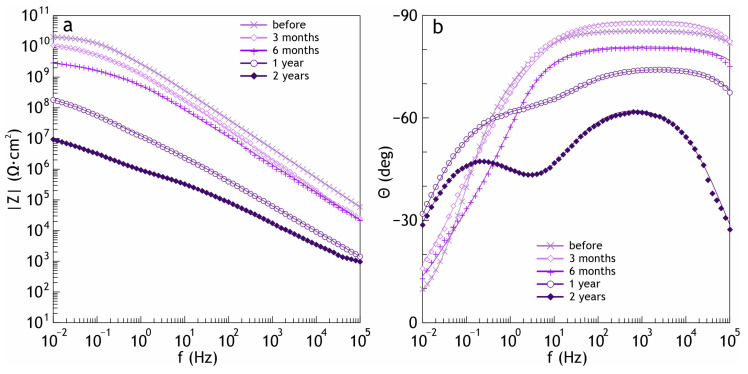
Evolution of the impedance modulus (**a**) and the phase angle (**b**) for PVDF:SPTFE 1:5 sample before and after 3 months, 6 months, 1 year, and 2 years of the atmosphere corrosion testing.

Electrochemical studies of samples after the atmosphere corrosion testing confirmed the abovementioned phenomenon. The potentiodynamic polarization curves confirmed that the PVDF:SPTFE 1:5 sample at the beginning of exposure was practically not subject to corrosion (Figure 12, Table S3). The increase in the corrosion current density was 1.5 orders of magnitude, given that its initial value was extremely low (7.5 × 10^−12^ A·cm^−2^). The impedance modulus remained almost at the same high level (1.6 × 10^10^ Ω·cm^2^), which confirms the high barrier properties after 3 months of exposure to a corrosive atmosphere (Figure 13, Table S4). At the same time, the increase in corrosion current indicates that microdefects, which are not visible in Figure 11d, were starting to form in this polymer layer. The value of the phase angle *Θ* for the PVDF:SPTFE 1:5 sample exhibited a capacitive character and tended to decrease to –90° at high and medium frequencies because of embedded SPTFE microparticles in the pores.

Analysis of potentiodynamic curves for the sample after two years of exposure shows that with the formation of microdefects in the polymer film (Figure 11h), the *I*_C_ value increased by almost three orders of magnitude (Figure 12).

Based on the analysis of the results of impedance spectroscopy of samples after 1 and 2 years of exposure, we can conclude that the dependence of the phase angle on frequency is similar to that of the PEO layer and has two time constants. This indicates that during this test period, there was a decrease in the corrosion resistance of the PVDF:SPTFE 1:5 sample.

## 4. Conclusions

As a result of the presented study, a combination of methods for tests of anticorrosion properties of the samples of aluminum alloy with different types of coatings was proposed.

Scanning electron microscopy and electrochemical techniques were used to study composite coatings obtained by applying combinations of PVDF and SPTFE polymers in ratios of 1:1 to 1:10 atop a PEO layer. Thus, we determined the ratio of PVDF to SPTFE equal to 1:5 as the “golden mean” and identified the reasons for the lower quality of coatings with a lower or higher ratio of polymers used:

❖ A decrease from this optimal ratio leads to nonuniformity of the surface relief created by SPTFE microparticles and a decrease in the thickness of the composite layer;
❖ An increase in the ratio leads to oversaturation of the PVDF film with SPTFE microparticles, deteriorating their binding to each other, which leads to defects (cracks) in the composite coating even at the stage of solvent evaporation.

After 40 days salt spray testing, the PVDF:SPTFE 1:5 sample demonstrated the lowest tendency for an increase in corrosion current density—less than one order of magnitude. But, in general, during the corrosion studies for fluorine-containing films with a PVDF:SPTFE ratio of 1:1–1:5, the formation of defects was not large.

As a result of two-year tests for atmospheric corrosion, it was revealed that the electrochemical parameters of the PVDF: SPTFE 1:5 sample remained consistently high during the year of exposure.

## Figures and Tables

**Table 1:** Designation of the studied AlMg3 aluminum alloy samples.

Sample	Treatment Condition			
	Plasma Electrolytic Oxidation	Dip-Coating		
		Dispersive Phase (SPTFE)	Dispersive Media	PVDF:SPTFE Ratio
Uncoated	–	–	–	–
PEO	+	–	–	–
PEO/PVDF	+	+	6% PVDF solution in N-methyl-2-pyrrolidone	–
PVDF:SPTFE 1:1	+	+		1:1
PVDF:SPTFE 1:2	+	+		1:2
PVDF:SPTFE 1:3	+	+		1:3
PVDF:SPTFE 1:4	+	+		1:4
PVDF:SPTFE 1:5	+	+		1:5
PVDF:SPTFE 1:10	+	+		1:10

**Table 2:** Calculated electrochemical parameters for studied samples.

Sample	*E*_C_ [V vs. Ag/AgCl]	*I*_C_ [A∙cm^−2^]	*R*_p_ [Ω∙cm^2^]
Uncoated	−0.67	1.1 × 10^−6^	2.4 × 10^4^
PEO	−0.87	8.4 × 10^−8^	1.9 × 10^5^
PEO/PVDF	–0.82	8.1 × 10^−11^	1.3 × 10^9^
PVDF:SPTFE 1:1	−0.53	7.2 × 10^−11^	2.7 × 10^9^
PVDF:SPTFE 1:3	−0.57	1.5 × 10^−11^	1.7 × 10^10^
PVDF:SPTFE 1:5	−0.59	7.5 × 10^−12^	3.9 × 10^10^
PVDF:SPTFE 1:10	−0.53	3.9 × 10^−10^	8.7 × 10^8^

**Table 3:** Calculated electrochemical parameters from Bode plot of uncoated and coated samples.

Sample	*CPE*_1_		*R*_1_ [Ω∙cm^2^]	*CPE*_2_		*R*_2_ [Ω∙cm^2^]	*|Z|*_f = 0.01 Hz_ [Ω∙cm^2^]
	*Q_1_* [Ω^−1^∙cm^−2^ s^n^]	*n*_1_		*Q*_2_ [Ω^−1^∙cm^−2^ s^n^]	*n*_2_		
Uncoated	9.21 × 10^−6^	0.91	3.11 × 10^4^	−	−	−	2.9 × 10^4^
PEO	6.08 × 10^−8^	0.88	2.48 × 10^4^	6.72 × 10^−7^	0.72	8.61 × 10^6^	5.6 × 10^6^
PEO/PVDF	6.19 × 10^−10^	0.89	1.14 × 10^9^	4.14 × 10^−9^	0.64	2.35 × 10^9^	1.9 × 10^9^
PVDF:SPTFE 1:1	1.68 × 10^−10^	0.97	3.19 × 10^8^	5.00 × 10^−10^	0.56	3.30 × 10^9^	3.0 × 10^9^
PVDF:SPTFE 1:3	7.31 × 10^−11^	0.98	3.53 × 10^8^	1.13 × 10^−10^	0.56	6.29 × 10^9^	5.9 × 10^9^
PVDF:SPTFE 1:5	5.34 × 10^−11^	0.95	4.88 × 10^9^	5.21 × 10^−11^	0.68	1.65 × 10^10^	2.0 × 10^10^
PVDF:SPTFE 1:10	1.92 × 10^−11^	0.98	7.14 × 10^9^	2.18 × 10^−11^	0.60	7.78 × 10^10^	5.4 × 10^7^

## Data Availability

The original contributions presented in the study are included in the article/Supplementary Material, further inquiries can be directed to the corresponding author.

## Supplementary Materials

The following supporting information can be downloaded at https://www.mdpi.com/article/10.3390/polym16202945/s1. Table S1: Calculated electrochemical parameters from polarization curves after 4, 10, 20, 30, and 40 days of salt spray testing of uncoated and samples with PEO and composite coatings; Table S2: Calculated electrochemical parameters from Bode plots after 4, 10, 20, 30, and 40 days of salt spray testing of uncoated and PEO, PEO/PVDF and PVDF:SPTFE 1:1–1:5 samples; Table S3: Calculated electrochemical parameters from polarization curves after atmospheric corrosion of uncoated and samples with PEO and composite coatings; Table S4: Calculated electrochemical parameters from Bode plots after atmospheric corrosion of uncoated and samples with PEO and composite coatings.
