# Supplementary material for: Corrosion Properties of the Composite Coatings Formed on PEO Pretreated AlMg3 Aluminum Alloy by Dip-Coating in Polyvinylidene Fluoride-Polytetrafluoroethylene Suspension
Source: Polymers (Basel). 2024 Oct 21;16(20):2945. doi: 10.3390/polym16202945 (PMC11511163; doi:10.3390/polym16202945)
Supplement: Supplementary file 1 [file polymers-16-02945-s001.zip › polymers-3244216-supplementary.pdf]

polymers

MDPI

Article

Corrosion Properties of the Composite Coatings Formed on PEO Pretreated AlMg3 Aluminum Alloy by Dip-Coating in Polyvinylidene Fluoride-Polytetrafluoroethylene Suspension

Vladimir S. Egorkin, Igor E. Vyaliy *, Andrey S. Gnedenkov, Ulyana V. Kharchenko, Sergey L. Sinebryukhov and Sergey V. Gnedenkov

Institute of Chemistry Far Eastern Branch of the Russian Academy of Sciences, Vladivostok 690022, Russia; egorkin@ich.dvo.ru (V.S.E.); asg17@mail.com (A.S.G.); ulyana-kchar@mail.ru (U.V.K.); sls@ich.dvo.ru (S.L.S.); svg21@hotmail.com (S.V.G.)

* Correspondence: igorvyal@gmail.com

Supplementary materials

The following data, not included in the article, obtained from the calculation of polarization curves (Figure 7 and Figure 12) and modeling of Bode diagrams (Figure 9 and Figure 13) are presented in this document on tables S1–S4.

Table S1. Calculated electrochemical parameters from polarization curves after 4, 10, 20, 30 and 40 days of salt spray testing of uncoated and samples with PEO and composite coatings

Sample	Test time	E_c [V vs. Ag/AgCl]	I_c [A·cm^{-2}]	R_p [Ω·cm^2]
Uncoated	4 days	–0.91	1.9×10^{-5}	2.9×10^{3}
	10 days	–1.01	2.5×10^{-5}	2.7×10^{3}
	20 days	–1.06	3.5×10^{-5}	1.9×10^{3}
	30 days	–1.09	3.9×10^{-5}	1.6×10^{3}
	40 days	–1.14	4.4×10^{-5}	9.9×10^{2}
PEO	4 days	–0.84	7.6×10^{-8}	1.0×10^{5}
	10 days	–0.81	8.3×10^{-8}	9.5×10^{4}
	20 days	–0.74	8.9×10^{-8}	9.1×10^{4}
	30 days	–0.71	9.6×10^{-8}	8.7×10^{4}
	40 days	–0.67	1.1×10^{-7}	6.4×10^{4}
PEO/PVDF	4 days	–0.81	3.4×10^{-10}	9.6×10^{7}
	10 days	–0.79	9.9×10^{-10}	2.0×10^{7}
	20 days	–0.77	1.9×10^{-9}	4.7×10^{6}
	30 days	–0.75	1.1×10^{-8}	3.8×10^{5}
	40 days	–0.70	1.4×10^{-8}	3.3×10^{5}
PVDF:SPTFE 1:1	4 days	–0.48	3.3×10^{-10}	8.8×10^{7}
	10 days	–0.41	3.9×10^{-10}	8.1×10^{7}
	20 days	–0.36	1.3×10^{-9}	5.5×10^{6}
	30 days	–0.32	2.8×10^{-9}	3.4×10^{6}
	40 days	–0.27	5.1×10^{-9}	1.2×10^{6}
PVDF:SPTFE 1:2	4 days	–0.34	3.1×10^{-10}	5.6×10^{8}
	10 days	–0.31	3.5×10^{-10}	5.0×10^{8}
	20 days	–0.27	4.0×10^{-10}	4.3×10^{8}
	30 days	–0.23	4.9×10^{-10}	3.6×10^{8}
	40 days	–0.17	6.1×10^{-10}	2.5×10^{8}

Continued **Table S1**:

PVDF:SPTFE 1:3	4 days	–0.49	3.5×10^{-11}	9.1×10^{9}
	10 days	–0.42	6.0×10^{-11}	6.3×10^{9}
	20 days	–0.36	6.1×10^{-11}	6.0×10^{9}
	30 days	–0.30	8.6×10^{-11}	4.2×10^{9}
	40 days	–0.30	1.8×10^{-10}	5.8×10^{8}
PVDF:SPTFE 1:4	4 days	–0.51	9.0×10^{-12}	3.4×10^{10}
	10 days	–0.44	1.9×10^{-11}	1.2×10^{10}
	20 days	–0.39	2.7×10^{-11}	9.0×10^{9}
	30 days	–0.37	4.9×10^{-11}	6.8×10^{9}
	40 days	–0.32	6.6×10^{-11}	4.7×10^{9}
PVDF:SPTFE 1:5	4 days	–0.51	1.1×10^{-11}	7.7×10^{9}
	10 days	–0.38	1.7×10^{-11}	7.1×10^{9}
	20 days	–0.31	2.8×10^{-11}	6.3×10^{9}
	30 days	–0.18	4.9×10^{-11}	3.6×10^{9}
	40 days	–0.09	6.0×10^{-11}	2.9×10^{9}

Table S2. Calculated electrochemical parameters from Bode plots after 4, 10, 20, 30 and 40 days of salt spray testing of uncoated and PEO, PEO/PVDF and PVDF:SPTFE 1:1–1:5 samples

**Sample**	**Test time**	***CPE₁*** Q_1 [$\Omega^{-1} \cdot cm^{-2}\, s^n$]	n_1	R_1 [$\Omega \cdot cm^2$]	***CPE₂*** Q_2 [$\Omega^{-1} \cdot cm^{-2}\, s^n$]	n_2	R_2 [$\Omega \cdot cm^2$]	$\lvert Z \rvert_{f=0.01\ Hz}$ [$\Omega \cdot cm^2$]
Uncoated	4 days	–	–	–	2.01×10^{-5}	0.83	5.68×10^{3}	5.5×10^{4}
	10 days	–	–	–	1.67×10^{-5}	0.82	4.79×10^{3}	4.1×10^{4}
	20 days	–	–	–	1.29×10^{-5}	0.81	3.74×10^{3}	3.6×10^{4}
	30 days	–	–	–	1.20×10^{-5}	0.78	3.28×10^{3}	3.3×10^{4}
	40 days	–	–	–	1.13×10^{-5}	0.77	2.68×10^{3}	2.7×10^{4}
PEO	4 days	6.13×10^{-8}	0.86	3.19×10^{4}	7.04×10^{-7}	0.73	1.01×10^{7}	6.1×10^{6}
	10 days	6.20×10^{-8}	0.87	2.80×10^{4}	6.85×10^{-7}	0.72	8.60×10^{6}	5.6×10^{6}
	20 days	5.89×10^{-8}	0.87	2.23×10^{4}	6.57×10^{-7}	0.69	7.89×10^{6}	5.0×10^{6}
	30 days	5.13×10^{-8}	0.88	1.78×10^{4}	5.89×10^{-7}	0.67	6.75×10^{6}	4.7×10^{6}
	40 days	5.01×10^{-8}	0.89	1.57×10^{4}	5.71×10^{-7}	0.65	6.54×10^{6}	4.5×10^{6}
PEO/PVDF	4 days	3.90×10^{-9}	0.77	4.37×10^{8}	2.34×10^{-8}	0.81	5.44×10^{7}	4.3×10^{8}
	10 days	9.09×10^{-9}	0.74	1.57×10^{8}	8.34×10^{-8}	0.76	1.94×10^{7}	1.6×10^{8}
	20 days	1.10×10^{-8}	0.72	4.51×10^{7}	3.66×10^{-7}	0.74	8.28×10^{6}	5.1×10^{7}
	30 days	1.39×10^{-8}	0.69	2.40×10^{6}	4.20×10^{-7}	0.71	7.40×10^{6}	8.3×10^{6}
	40 days	3.48×10^{-8}	0.59	1.10×10^{6}	5.13×10^{-7}	0.69	6.13×10^{6}	5.7×10^{6}
PVDF:SPTFE 1:1	4 days	3.67×10^{-10}	0.93	6.31×10^{6}	8.90×10^{-10}	0.55	4.60×10^{8}	4.4×10^{8}
	10 days	5.41×10^{-9}	0.90	4.91×10^{6}	9.90×10^{-10}	0.50	3.84×10^{8}	3.5×10^{8}
	20 days	7.69×10^{-10}	0.84	1.11×10^{6}	2.98×10^{-8}	0.50	6.06×10^{7}	4.7×10^{7}
	30 days	2.39×10^{-9}	0.76	8.19×10^{5}	3.99×10^{-8}	0.48	3.88×10^{7}	3.0×10^{7}
	40 days	4.74×10^{-9}	0.76	2.61×10^{5}	8.99×10^{-8}	0.48	1.20×10^{7}	9.4×10^{6}
PVDF:SPTFE 1:2	4 days	3.07×10^{-10}	0.95	9.91×10^{7}	9.30×10^{-10}	0.57	2.99×10^{8}	3.8×10^{8}
	10 days	4.27×10^{-10}	0.92	6.19×10^{7}	1.09×10^{-9}	0.52	2.19×10^{8}	2.8×10^{8}
	20 days	6.30×10^{-10}	0.91	4.23×10^{7}	3.79×10^{-9}	0.47	9.45×10^{7}	1.3×10^{8}
	30 days	8.21×10^{-10}	0.89	3.21×10^{7}	5.72×10^{-9}	0.40	7.34×10^{7}	1.0×10^{8}
	40 days	9.33×10^{-10}	0.88	2.14×10^{7}	8.81×10^{-9}	0.36	5.57×10^{7}	6.8×10^{7}

Continued **Table S2**:

PVDF:SPTFE 1:3	4 days	7.79 × 10^{-11}	0.96	3.03 × 10^{8}	1.69 × 10^{-10}	0.50	4.28 × 10^{9}	4.3 × 10^{9}
	10 days	8.47 × 10^{-11}	0.95	1.06 × 10^{8}	2.55 × 10^{-10}	0.48	2.09 × 10^{9}	1.9 × 10^{9}
	20 days	9.39 × 10^{-11}	0.94	9.26 × 10^{7}	3.44 × 10^{-10}	0.47	1.09 × 10^{9}	1.0 × 10^{9}
	30 days	1.24 × 10^{-10}	0.93	6.06 × 10^{7}	5.55 × 10^{-10}	0.41	6.99 × 10^{8}	6.8 × 10^{8}
	40 days	2.22 × 10^{-10}	0.89	4.11 × 10^{7}	7.94 × 10^{-10}	0.39	3.49 × 10^{8}	3.7 × 10^{8}
PVDF:SPTFE 1:4	4 days	7.30 × 10^{-11}	0.94	6.49 × 10^{8}	1.11 × 10^{-10}	0.60	9.89 × 10^{9}	9.2 × 10^{9}
	10 days	9.93 × 10^{-11}	0.92	3.95 × 10^{8}	1.90 × 10^{-10}	0.56	6.14 × 10^{9}	5.4 × 10^{9}
	20 days	1.01 × 10^{-10}	0.91	3.10 × 10^{8}	2.23 × 10^{-10}	0.50	5.27 × 10^{9}	4.6 × 10^{9}
	30 days	1.70 × 10^{-10}	0.89	1.05 × 10^{8}	4.93 × 10^{-10}	0.49	2.67 × 10^{9}	2.1 × 10^{9}
	40 days	2.69 × 10^{-10}	0.86	9.47 × 10^{7}	5.73 × 10^{-10}	0.47	1.47 × 10^{9}	1.4 × 10^{9}
PVDF:SPTFE 1:5	4 days	8.14 × 10^{-11}	0.94	3.89 × 10^{9}	2.40 × 10^{-10}	0.65	6.80 × 10^{9}	9.5 × 10^{9}
	10 days	9.05 × 10^{-11}	0.93	3.51 × 10^{9}	3.97 × 10^{-10}	0.61	5.29 × 10^{9}	7.4 × 10^{9}
	20 days	9.85 × 10^{-11}	0.92	2.63 × 10^{9}	5.22 × 10^{-10}	0.59	3.97 × 10^{9}	5.5 × 10^{9}
	30 days	1.80 × 10^{-10}	0.90	1.30 × 10^{9}	7.08 × 10^{-10}	0.51	2.33 × 10^{9}	3.0 × 10^{9}
	40 days	2.58 × 10^{-10}	0.88	9.11 × 10^{8}	8.22 × 10^{-10}	0.50	1.41 × 10^{9}	2.1 × 10^{9}

Table S3. Calculated electrochemical parameters from polarization curves after atmospheric corrosion of uncoated and samples with PEO and composite coatings

Sample	Test time	E_c [V vs. Ag/AgCl]	I_c [A·cm^{-2}]	R_p [Ω·cm^2]
Uncoated	3 months	–0.73	1.6 × 10^{-6}	2.5 × 10^{3}
	6 months	–0.95	1.7 × 10^{-5}	3.8 × 10^{2}
	1 year	–1.04	5.5 × 10^{-5}	9.8 × 10^{1}
	2 years	–1.19	6.3 × 10^{-5}	4.9 × 10^{1}
PEO	3 months	–0.79	6.9 × 10^{-8}	4.3 × 10^{5}
	6 months	–0.69	3.5 × 10^{-8}	5.7 × 10^{5}
	1 year	–0.64	6.3 × 10^{-8}	3.5 × 10^{5}
	2 years	–0.71	3.1 × 10^{-7}	6.6 × 10^{4}
PEO/PVDF	3 months	–0.80	2.9 × 10^{-10}	8.9 × 10^{7}
	6 months	–0.78	4.8 × 10^{-9}	9.7 × 10^{6}
	1 year	–0.73	3.3 × 10^{-9}	8.6 × 10^{5}
	2 years	–0.69	1.4 × 10^{-8}	1.0 × 10^{5}
PVDF:SPTFE 1:5	3 months	–0.53	1.5 × 10^{-11}	4.9 × 10^{9}
	6 months	–0.48	2.9 × 10^{-11}	2.3 × 10^{9}
	1 year	–0.46	2.7 × 10^{-10}	8.0 × 10^{7}
	2 years	–0.79	6.7 × 10^{-9}	5.5 × 10^{6}

Table S4. Calculated electrochemical parameters from Bode plot after atmospheric corrosion of uncoated and samples with PEO and composite coatings

Sample	Test time	*CPE$_1$*		*R_1 [Ω·cm^2]*	*CPE$_2$*		*R_2 [Ω·cm^2]*	*$\|Z\|_{f=0.01 Hz}$ [Ω·cm^2]*
		Q_1 [Ω^{-1}·cm^{-2} s^n]	*n_1*		*Q_2 [Ω^{-1}·cm^{-2} s^n]*	*n_2*		
Uncoated	3 months	–	–	–	1.36 × 10^{-5}	0.88	1.98 × 10^{4}	2.0 × 10^{4}
	6 months	–	–	–	1.10 × 10^{-5}	0.89	7.26 × 10^{3}	7.3 × 10^{3}
	1 year	–	–	–	1.00 × 10^{-5}	0.88	3.24 × 10^{3}	3.3 × 10^{3}
	2 years	–	–	–	1.10 × 10^{-5}	0.84	1.79 × 10^{3}	1.8 × 10^{3}

Continued **Table S4**:

PEO	3 months	2.84×10^{-8}	0.74	1.91×10^{5}	4.88×10^{-7}	0.60	9.07×10^{6}	5.2×10^{6}
	6 months	3.02×10^{-8}	0.72	3.46×10^{5}	4.83×10^{-7}	0.69	5.49×10^{6}	4.0×10^{6}
	1 year	9.30×10^{-7}	0.64	1.91×10^{5}	6.63×10^{-7}	0.65	1.45×10^{6}	1.3×10^{6}
	2 years	1.51×10^{-5}	0.61	9.50×10^{4}	7.63×10^{-7}	0.65	5.39×10^{5}	5.5×10^{5}
PEO/PVDF	3 months	9.69×10^{-10}	0.87	9.16×10^{8}	1.58×10^{-8}	0.59	5.17×10^{9}	1.0×10^{9}
	6 months	1.97×10^{-9}	0.83	4.59×10^{8}	6.58×10^{-8}	0.55	2.19×10^{8}	4.9×10^{8}
	1 year	2.09×10^{-8}	0.70	2.96×10^{7}	9.91×10^{-8}	0.54	9.93×10^{6}	3.6×10^{7}
	2 years	1.29×10^{-7}	0.59	1.06×10^{5}	5.61×10^{-7}	0.50	1.33×10^{6}	1.3×10^{6}
PVDF:SPTFE 1:5	3 months	9.23×10^{-11}	0.98	7.41×10^{8}	1.49×10^{-10}	0.53	1.36×10^{10}	1.6×10^{10}
	6 months	2.77×10^{-10}	0.90	8.91×10^{8}	8.01×10^{-10}	0.58	2.60×10^{9}	2.9×10^{9}
	1 year	1.11×10^{-8}	0.83	5.11×10^{6}	1.25×10^{-8}	0.60	3.22×10^{8}	1.8×10^{8}
	2 years	1.09×10^{-7}	0.73	6.01×10^{5}	3.23×10^{-7}	0.63	1.51×10^{7}	9.3×10^{6}
